# Latin America and access to Assisted Reproductive Techniques: A Brazilian perspective

**DOI:** 10.5935/1518-0557.20140004

**Published:** 2014

**Authors:** Maria do Carmo B. de Souza

**Affiliations:** 1 REDLARA - The Latin American Network of Assisted Reproduction; 2 Fertipraxis - Human Reproduction - RJ

**Keywords:** Asynchronic delivery, PROM, delay delivery, triplet pregnancy

## Abstract

**Introduction:**

In Brazil, as in all Latin America, access to infertility care, including assisted reproductive technology (ART) is on debate. This paper evaluates the availability and access of Brazilian couples to ART services.

**Methods:**

It is a qualitative study conducted about the Brazilian possibilities of ART in the public sector. A questionnaire was sent by e-mail to 14 public centers identified as providing ART (IVF/ICSI and/or IUI), with questions about their performance on 2013. The questionnaire was objective with seven questions. It was asked the number of patients seen for 1^st^ time in 2013, and number IVF procedures and/or IUI in this interval, the source of funds to support the center, the input source of the patients, who pays for medications and how much (%), number of cycles or age limitations, whether or not oocyte donation is held.

**Results:**

There were 11 answers out of 14 and during 2013 in public centers were performed 1088 IUI cycles plus 4044 IVF/ICSI cycles. The waiting lists of patients can vary from 300 to 1500 and wait from 6 months to 4 years.

**Conclusions:**

As infertility goes, charges remain incompatible with the financial possibilities of the majority of Brazilian population. The Brazilian government should consider buying cycles in private accredited centers to offer ART procedures at no cost to low-income populations. Other possibility is to state as mandatory that Health Insurance Assistance Companies cover ART treatments, making it accessible for a large part of the population.

## INTRODUCTION

How efficient are Health Public Systems in securing ART in Brazil nowadays? It is very well known that infertility can lead to marital breakdown, physical violence, emotional abuse and social exclusion. Consequently, access to the diagnosis and treatment of infertility, including ART, contributes to resolving social inequities and emotional difficulties (Boivin *et al*, 2007).

### Fertility in Brazil

If Latin America is not uniform either culturally or socially (UNDP/UNFPA/WHO, 2003), the same applies if we consider Brazil alone, which represents today 5 different regions (north, northeast, middle-west, southeast and south) with 200 hundred million people. There are great diversities among them and most part of the population and the highest income “per capita” are in the south and southeast areas (56% of population, IBGE 2010).

Although many Brazilians still share a strong statement of being catholic (64% IBGE 2010) there is not any more such a consequent influence of the catholic church on family planning decisions. In Brazil, the fertility rate has remained virtually constant from 1930 until 1965, when it began to decline. The total fertility rate (TFT) dropped from 5.8 children in 1970 (Ipea, 1996) to 1.82 in 2012 (UN Data, 2012).

The population control achieved was not effective in reducing poverty and women commonly include maternity as an inherent and necessary condition for achieving their potential (Costa *et al*, 2006). Pregnancy and maternity are experienced not only as a bodily process, but also as the assignment of a superior status to women in relation to those without children (Paim,1998). The PNAD (National Survey through Home Sampling) results from 2007 show women over forty years, who are mothers for the first time, have financial stability and higher levels of education, showing the deep inequality of the Brazilian reality with regard to reproductive health.

Therefore, issues related to infertility arouse strong emotions and are related to low self-esteem, isolation, fear, lack of social status and even violence. On false premises of overpopulation, infertility people have been considered a non-priority group in Public Health Assistance, which means that social damage is maintained (Fathalla, 2002).

“The current forms of medical organization and the complexity of the associated technology... in many cases withdraw, or increase the withdrawal of, the participation of common people from decision-making regarding their own body, for their well-being and ultimately, the fate of their lives”. (Correa, 2001).

Assisted reproduction, according to these people, is frequently considered a goal to achieve and for this couples (and women, mostly) would go to great lengths to have the possibilities that “rich people have”. This lack of empowerment besides low self-esteem certainly exposes low income people to either unclear or unfair situations of gamete donation and surrogacy (Lopes, 2008). When they have not succeeded in their attempts at private centers or because they cannot pay for them, these people arrive at public health units declaring feelings of resentment and discrimination.

### Political definitions: a gap between regulations and reality

Federal Council of Medicine - Resolution CFM 1358/1992 - states about general rules for ART procedures, followed by CFM 1957/2010 and now CFM 2013/2013.

SUS - The Brazilian Health System, universal free medical assistance guaranteed by the Constitution of 1998.

Politica Nacional de Atenção Integral em Reprodução Humana Assistida, SUS. Portaria 426, 22/03/2005. Instructions relating to the policies of implementation of primary, secondary and tertiary centers of ART in the country.

Lei de Biossegurança - 11/03/2005, deals with frozen embryos and their final destination. Stem cell research.

RDC 33, 2006 (ANVISA- National Surveillance Agency) regulates ART and minimum conditions for ART labs in the country. A new contribution in RDC 23, 2011.

TFD - (Out of home state treatments) Portaria SAS/055 de 24/02/99; TFD RJ DO 179, 20/09/2002. Legal instrument that guarantees specialized assistance for diseases not treatable in the area where the patient lives (high and medium complexity). It is supposed to offer consultation, treatment, money for travelling, accommodation and stipend (US$75 per person, each time). The state of Rio de Janeiro has an amount for these situations per year and ART was the third request in 2010. There is no available report at all of results obtained in ART.

## MATERIALS AND METHODS

At the end of 2012 the Ministry published the Order n.3.149/2012 that allocated resources and qualified 8 hospitals to perform procedures of assisted human reproduction within the SUS. These centers received a specific amount to handle services that were being run. Another 6 were identified through general press or personal knowledge and all these centers received a direct questionnaire (sent either to the center or to a professional that could answer or direct the e-mail to someone that could do it properly) asking information about their performance in the year 2013. All the questionnaires were sent by e-mail, at least 5 times, at one month interval, to: Centro de Ensino e Pesquisa em Reprodução Assistida (CEPRA), Brasilia DF; Centro de Referência em Saúde da Mulher - Hospital Pérola Byington, São Paulo, linked to the Department of Health of SP; Setor Integrado de Reprodução Humana da Escola Paulista de Medicina - UNIFESP - Hospital São Paulo, Centro de Reprodução Governador Mario Covas - Hospital das Clínicas (USP), São Paulo; Serviço de Reprodução Humana- Hospital das Clínicas de Ribeiro Preto (USP-RP) Ribeirão Preto, SP; Laboratório de Reprodução Humana Prof Aroldo Camargos, do Hospital de Clinicas da UFMG, Belo Horizonte, MG; Hospital Fêmina, Grupo Hospitalar Conceição, Porto Alegre (RS); Centro Hospital de Clinicas de Porto Alegre - Universidade Federal do Rio Grande do Sul (UFRGS); Instituto de Medicina Integral Prof. Fernando Figueira, em Recife (PE), Laboratório de Reprodução Humana - HC Goiás / UFG - LABREP (GO), Instituto Idéia Fertil de Saúde Reprodutiva - Faculdade de Medicina do ABC (SP), Maternidade Escola Januário Cicco (UFRN), Natal (RN); Hospital Alvaro Alvim, in Campos (RJ) and Instituto de Ginecologia da UFRJ, Rio de Janeiro (RJ). When no answer was obtained we also tried phone contacts and social networks like facebook. The questionnaire was objective with seven questions. It asked the number of patients seen for the first time in 2013, the number of IVF procedures and/or IUI in this interval, the source of funds to support the center, the source of the patients, who pays for medications and how much (%), number of cycles or age limitations and whether or not oocyte donation is offered.

## RESULTS

There were 11 answers out of 14 (Tables 1 and 2), sent respectively by Rosaly Rulli Costa, Centro de Ensino e Pesquisa em reprodução assistida (CEPRA), Brasilia DF; Mario Cavagna Neto, Centro de Referência em Saúde da Mulher - Hospital Pérola Byington, Eduardo Motta, Setor Integrado de Reprodução Humana da Escola Paulista de Medicina - UNIFESP ,Pedro Augusto Araujo Monteleone, Centro de Reprodução Governador Mario Covas (USP); Ana Carolina Japur de Sá Rosa e Silva, Serviço de Reprodução Humana - Hospital das Clínicas de Ribeiro Preto (USP-RP) Ribeirão Preto, SP; Francisco de Assis Nunes Pereira, Laboratório de Reprodução Humana Prof Aroldo Camargos, Eduardo Pandolfi Passos, Centro Hospital de Clinicas de Porto Alegre - Universidade Federal do Rio Grande do Sul (UFRGS); Mario Approbato, Laboratório de Reprodução Humana - HC Goiás / UFG - LABREP (GO), Caio Parente Barbosa, Instituto Idéia Fertil de Saúde Reprodutiva - Faculdade de Medicina do ABC (SP), Gabrielle Hernandes Vieira, Hospital Alvaro Alvim, in Campos (RJ) and Tonia Costa, Instituto de Ginecologia da UFRJ, Rio de Janeiro, RJ. There came no answers from Instituto de Medicina Integral Prof. Fernando Figueira, em Recife (PE), Hospital Fêmina, Grupo Hospitalar Conceição, Porto Alegre (RS) and Maternidade Escola Januario Cicco (RN).

**Table 1:** ART Health Public Services in Brazil General results from 11 public centers that hold IVF-ICSI and IUI in Brasil- 2013

Public Center	Region	n IUI	n IVF	Source of funds%				Imput of patients %	
				**P**	**R**	**D**	**Patients**	**SUS**	**Open Access**
CEPRA*	MW	21	99	100				100	
HPBSP	SE	11	359	100				100	
UNESP	SE	100	400	70			30		100
USP-SP*	SE	133	324	90			10	100	
USP-RP*	SE	53	447	80			20	100	
ABC-SP	SE	433	1758	2	5	3	90		100
UFRGS*	S	0	240	100				100	
FMG	MW	87	103	100				100	
UFMG*	SE	193	200	80	20			100	
HAA-RJ	SE	0	114	100				30	70
IG-UFRJ	SE	57	0	100					

SE southeast, S south, MW middle-west.

Source of funds - P public, R research, D donations, patients - direct payment

*Qualified with extra funds by SUS - Sistema Unico de Saude - Brasil

USP-RP and CEPRA are accredited REDLARA centers

Open access means a medical referral is not necessary

**Table 2:** General results from 11 public centers that hold IVF-ICSI and IUI in Brasil - 2013 Access to drugs, age and limit of cycles - oocyte donation

Public Center	payment drugs%				age			n cycles	Ooc donation	
	0	25	50	100	≤35	≤38	<40 <41		**yes**	**no**
CEPRA	x				unlimited			2	x	
HPBSP	x					x		1	x	
UNESP			x			x	30	unlimited	x	
USP-SP	x			x		x	10	2		x
USP-RP				x		x	20	unlimited		x
ABC-SP				x	unlimited			unlimited	x	
UFRGS					x			3		x
FMG			x				x	unlimited		x
UFMG	x						x	3		x
HAA-RJ	x				unlimited			ulimited		x
IG-UFRJ			x							

* CEPRA and USP-RP are Accredited REDLARA center

Tables 1 and 2 summarizes the answers.

## DISCUSSION

In Brazil nowadays there are around 47 million women of reproductive age (15-49 years of age) and this means at least four million infertile couples (IBGE, 2010), an overlooked and under-studied problem. If we add men, these figures can easily double.

The fact is that although services in ART can be expensive in developing Latin America (LA), to most couples this has not prevented its spread. LA has today a very efficient Registry (REDLARA), but it still lacks data. It is supposed to cover about 80% of the cycles and the main clinics, all of them open to checking and sharing data. REDLARA Registry had 145 clinics reporting 41232 ART cycles in 2011 (for the second time the data individually collected in a case-b y- case modality). Brazil, with 54 clinics at that time represented 46% of them (18952 cycles). Nowadays there are 167 centers in REDLARA, 61 from Brazil. It is not known how many centers there are in the whole country now but there are different estimates (IFFS Surveillance, 2013; Zegers-Hochschild *et al*., 2013) from 120-200 centers, mainly private ones. The Brazilian Society of Assisted Reproduction (SBRA) lists 118 in its site but the associates are individual members, not clinics, so they need not declare a center and it is not known if some of the nominated centers have their own lab. The official government agency reported mandatory data from 91 centers with laboratories in 2012 (Sisembrio, 2012).

Monthly medium income of economically active people in the main metropolitan areas of Brazil was around US 950 per capita in Dec 2013 (IBGE, 2013). So, to those who inevitably raise questions about whether ART services should be justifiable in a public health program, the answer is yes. They are meant to be there, safe and efficient and should be free from charges, as that is in the constitution of the SUS (Sistema Unico de Saude - Health Public Assistance).

If we take a look over the identified Public Services of ART in Brazil, we can identify that they represent specialists devoted to providing ART, mostly at University centers. However, their work still depends for support on cyclic political issues.

They have waiting lists of patients that can vary from 300 to 1500 and wait from 6 months to 4 years. Hospital Pérola Byington in SP did not open for new patients in 2013 in order to diminish this waiting list. In fact, during this year they could also apply funds received from the government to amplify the facility, as stated by Dr Mario Cavagna. LABREP in Goiás also closed for 2 months during 2013 while finishing a new center that resulted from university extension projects and a designated personal political budget, as declared Dr Mario Approbato.

The public units in Brazil that provide ART are centered in the Southeast, South and Middle-West (Makuch *et al*, 2011). This is also clear in table 1 but regrettably the 2 official public ART centers from the Northeast did not answer the questionnaire. Every public center is meant to receive patients from regional units, from the same town or state. However, it is not uncommon that couples will try to come from other states in the country because they have no options there.

Having been asked to state the profile of their patients, it was not possible to have an exact number of first appointments per month of patients for infertility in each center. Most sent the number of patients just for ART. Some declared they do not have limits for women’s age or number of cycles (table 2), while others accept until 35 years as an access limit, <38 years (4 centers) or they can extend as far as 40- 41 years old. Goias (Middle West) does not treat women with BMI >31 or with 3 or more previous C-sections. Hospital Alvaro Alvim states that a womans age is always a priority, but this did not prevent them from answering unlimited access for age or number of cycles.

This lack of clear definition of criteria for acceptance probably contributes to the waiting lists. And patients can get older on the waiting list (Leite *et al*, 2011) without being in a financial position to carry out the treatment in the private sector or in other states. This results in exaggerating the length of these waiting lists, which also dooms many attempts to failure.

It is not uncommon now that Justice demands an intervention in the queues, as stated by Eduardo Passos (UFRGS). These conditions that drive patients to call for Justice in order to be granted public treatment is being called the judicialization of health. “This unique characteristic of health leads to approaches to the judiciary and the suffering population needs assistance” (Bahia, 2014).

In just 5 centers patients receive a full no-cost treatment. Most public center patients have to pay for their protocols. It is then that some private initiatives help, as is the case of “Programa Acesso”, when the drug costs reduce by around 50%. It has the support of Merck Serono. This manufacturer of some of the biochemical drugs currently used in the ART protocols offer the program in 56 Brazilian cities. In the selection criteria the couple must prove a certain gross annual income or be included in a public hospital program. Data from them, from Jan 2012 to Dec 2013, show that 67% of the requests were approved and 78% of the patients underwent procedures.

In total during 2013 there resulted 1088 IUI cycles plus 4044 IVF/ICSI cycles performed in public centers related to SUS. Some centers, such as UFRGS and Hospital Alvaro Alvim, did no IUI procedures. The former made a decision to focus on high complexity ART, while the latter made an effort to use INVO procedures to keep the costs down (Coelho *et al*, 2013). UFRJ described only IUI and did not return other details of its program in 2013.

Instituto Ideia Fertil Reprodutiva, from Faculdade de Medicina do ABC was responsible for the biggest contribution of cycles. In fact it is a Civil Society Organization of Public Interest (OSCIP), declared Dr Caio Parente Barbosa, supported by 90% of patients paying the center. It receives 2% of public funds to treat couples with chronic viral diseases and there is no charge for drugs to oncological patients.

Is this enough? The answer is certainly no.

Working towards social welfare, tolerance, cooperation and multiculturalism is perhaps one of the possible ways for the installation of a real reproductive right, in which the notion of equity would refer to inclusion and to each gender. As pointed by Garcia (2012), it is essential to deepen the discussion about the use and impact of ART, facing the social, ethical and legal challenges.

In November 1997, the Ministry of Health regulated the implementation of sterilization services in the Public Health System (SUS) - to be paid by the government and that was an example of national public policy profoundly influenced by the women’s movement. Now we have to build a similar path towards ART to guarantee the allocation of more resources.

Brazil is supposed to stabilize its population in 2030 and it will probably begin declining from 2050 on (Alves, 2013). As the population grows older, who will maintain social security and development? Developed countries have already faced that question.

To overcome this situation it is mandatory that Societies like REDLARA, and national societies as SBRA (Brazilian Society of Assisted Reproductive), SBRH (Brazilian Society of Human Reproduction) and Febrasgo (Brazilian Federation of Obstetrics and Gynecology Societies) build and spread Educational Programs in Reproductive Rights, Choices and ART. They have to guide not only doctors and patients, but it is their mission to expand their view of these issues. In doing this, the attention of Public Managers will be directed to these issues.

Private practice is not menaced by this demand. On the contrary, possible solutions include them. The Brazilian government could consider buying cycles in private accredited centers to offer ART procedures to low-income populations. The Argentinian experience could probably be a starting point to check (Argentina. ar, 2013). Again, lower cost programs could be defined within clear limits and expectations of success.

Another possibility is to state as mandatory that plans for Health Care cover ART treatments that would make it affordable to another considerable part of the population because WHO has already defined Infertility as a disease (Zegers-Hochschild *et al*, 2010). The Board of REDLARA is working throughout Latin America, inviting centers to join the organization. Public centers identified now will receive an invitation to become affiliated to REDLARA. And an active search of the exact number of centers (laboratories) in the country begins. An adequate follow-up of cases and cooperation among centers will enhance both Brazil and Latin American visibility and even more robust data will help governments to design public policies.

## CONCLUSION

Infertility charges remain incompatible with the financial possibilities of the majority of the Brazilian population. The Brazilian government should consider buying cycles in private accredited centers to offer ART procedures at no cost to low-income populations. Another possibility is to state as mandatory that Health Insurance Assistance Companies cover ART treatments, making it accessible for a large part of the population.

## References

[r1] Alves JED Crescimento populacional zero no Brasil no século XXI. http://www.ie.ufrj.br/aparte/pdfs/crescimento_populacional_zero_no_brasil_no_seculo_xxi.pdf.

[r2] Argentina.ar Portal público de noticias Ley de Fertilización Assistida: la reglamentación., 23/07/2013.

[r3] Bahia L. A judicialização da saúde.

[r4] Boivin J, Bunting L, Collins JA, Nygren KG (2007). International estimates of infertility prevalence and treatment-seeking: potential need and demand for infertility medical care. Hum Reprod.

[r5] CFM - Conselho Federal de Medicina (Federal Council of Medicine) Resolução 1382/92 http://www.portalmedico.org.br/resolucoes/cfm/1992/1358_1992.htm.

[r6] CFM - Conselho Federal de Medicina (Federal Council of Medicine) Resolução 1957/2010 http://www.portalmedico.org.br/resolucoes/CFM/2010/1957_2010.htm.

[r7] CFM - Conselho Federal de Medicina Resolução (Federal Council of Medicine) Resolução 2013/2013.

[r8] Coelho F, Aguiar LF, Cunha GSP, Lucena E (2013). Introduction of the method of intravaginal culture (IVC), through the device InVOCell routine laboratory RHA in Brazil. JBRA Assist. Reprod.

[r9] Correa MV (2001). Novas tecnologias reprodutivas: limites da biologia ou biologia sem limites?.

[r10] Costa T, Stotz E, Grynszpan D, Souza MCB (2006). Naturalization and medicalization of the female body: social control through reproduction. Interface-Comunic, Saude, Educ.

[r11] Fathalla MF, Vayena E, Rowe PJ, Griffin PD (2002). Current practice and controversies in assisted reproduction.

[r12] Garcia S (2012). Considerações sobre a reprodução assistida no contexto brasileiro.

[r13] IBGE - Instituto Brasileiro de geografia e Estatística - Brasil Censo Demografico 2010.

[r14] IBGE - Instituto Brasileiro de geografia e Estatística - Brasil Pesquisa mensal de empregos, Dezembro de 2013.

[r15] Ory SJ, Devroye P, IFFS Surveillance (2013). International Federation of Fertility Societies.

[r16] Lei de Biossegurança-11/03/2005 Brasil.

[r17] Leite TH, Silva WJJ, Moura SL, Costa T, Correa M, Souza MCB (2011). [Aging in Line for Artificial Insemination: The Reality of Infertile Couples in Low-Income from Rio de Janeiro]. JBRA Assist. Reprod.

[r18] Lopes AD Gravidez a soldo. Revista VEJA 7/5/2008.

[r19] Makuch AY, Padua KS, Petta CA, Osis MJD, Bahamondes L (2011). Inequitable access to assisted reproductive technology for the low-income Brazilian population: a qualitative study. Hum Reprod.

[r20] PNAD - National Survey through Home Sampling IBGE (2007). http://www.ibge.gov.br/home/estatistica/populacao/trabalhoerendimento/pnad2007/graficos_pdf.pdf.

[r21] Paim HHS, Duarte LFD, Leal OF (1998). Doença, sofrimento, perturbação: perspectivas etnográficas.

[r22] Politica Nacional de Atenção Integral em Reprodução Humana Assistida, Portaria 426, 22/03/2005.

[r23] Programa Acesso.

[r24] RDC 33 / ANVISA http://www.saude.mg.gov.br/atos_normativos/legislacao-sanitaria/estabelecimentos-de-saude/atencao-em-reproducao-humana-assistida-1/RDC_33.pdf.

[r25] RDC 23 /ANVISA http://portal.anvisa.gov.br/wps/wcm/connect/623ecb0047458efe9836dc3fbc4c6735/RDC_09_2011.pdf?MOD=AJPERES.

[r26] SisEmbrio: 6º Relatório do Sistema Nacional de Produção de Embriões.

[r27] SUS from A to Z.

[r28] TFD-RJ Resolução SES-RJ nº 171, de 28 de novembro de 2011.

[r29] UNDP/UNFPA/WHO/ World Bank Special Programme of research, Development and research training in Human Reproduction (HRP) (2003). Assisted reproduction in developing countries- facing up to the issues. Progress in Reproductive Health Research, n63.

[r30] (2012). UN Data, record view, Total Fertility Rate.

[r31] Zegers-Hochschild F, Adamson GD, De Mouzon J, Ishihara O, Mansour R, Nygren K, Sullivan E, Van Der Poel S (2010). Glossário revisado da Terminologia das Técnicas de Reprodução Assistida (TRA), 2009†, Comitê Internacional para Monitorização da Tecnologia Reprodutiva Assistida (ICMART) e Organização Mundial da Saúde (OMS). JBRA Assist. Reprod.

[r32] Zegers-Hochschild F, Schwarze JE, Crosby JA, Musri C, Souza MCB (2013). Assisted reproductive technologies (ART) in Latin America: The Latin American Registry 2011. JBRA Assist. Reprod.

